# Crystallographic analysis of the laminin β2 short arm reveals how the LF domain is inserted into a regular array of LE domains

**DOI:** 10.1016/j.matbio.2016.06.006

**Published:** 2017-01

**Authors:** David Pulido, David C. Briggs, Jinwen Hua, Erhard Hohenester

**Affiliations:** Department of Life Sciences, Imperial College London, UK

**Keywords:** Laminin, Basement membrane, X-ray crystallography

## Abstract

Laminins are a major constituent of all basement membranes. The polymerisation of laminins at the cell surface is mediated by the three short arms of the cross-shaped laminin heterotrimer. The short arms contain repeats of laminin-type epidermal growth factor-like (LE) domains, interspersed with globular domains of unknown function. A single LF domain is inserted between LE5 and LE6 of the laminin β1 and β2 chains. We report the crystal structure at 1.85 Å resolution of the laminin β2 LE5-LF-LE6 region. The LF domain consists of a β-sandwich related to bacterial family 35 carbohydrate binding modules, and more distantly to the L4 domains present in the short arms of laminin α and γ chains. An α-helical region mediates the extensive interaction of the LF domain with LE5. The relative arrangement of LE5 and LE6 is very similar to that of consecutive LE domains in uninterrupted LE tandems. Fitting atomic models to a low-resolution structure of the first eight domains of the laminin β1 chain determined by small-angle X-ray scattering suggests a deviation from the regular LE array at the LE4–LE5 junction. These results advance our understanding of laminin structure.

## Highlights

• The crystal structure of the laminin β2 LE5-LF-L6 region has been determined.
• The LF domain is a β-sandwich with distant homology to the L4 domains in laminin α and γ chains.
• A unique α-helical region in the LF domain interacts extensively with LE5.
• LE5 and LE6 are arranged in a manner typical of tandem LE domains, despite the insertion of the LF domain.

## Introduction

**Fig. 1:**
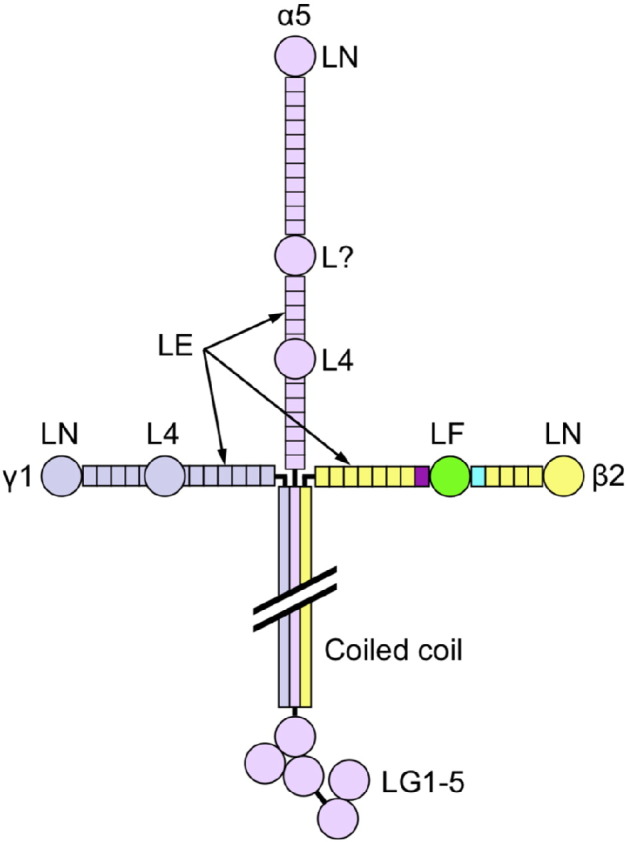
Domain structure of laminin-521. The crystallised β2 chain fragment is highlighted by colouring (LE5, cyan; LF, green; LE6, purple). A domain of unknown structure in the α5 chain is labelled “L?” (see text).

Heterotrimeric (αβγ) laminins are a major constituent of all basement membranes [1], [2]. They are present in even the simplest animals and presumed to be essential for multicellular life [3], [4]. In mammals, at least 16 laminins are assembled from one of five α chains, one of three β chains, and one of three γ chains [5]. The three chains associate through an α-helical coiled coil, the so-called long arm (80 nm length). At the N-terminus of the long arm, the three chains separate and form three distinct short arms (35–50 nm length), lending the laminin heterotrimers their characteristic cross-shaped appearance in electron micrographs (Fig. 1). In the α3A, α4, β3 and γ2 chains, the short arms are truncated or altogether absent. At the C-terminus of the long arm, the α chain continues for another ~ 1000 residues, which are folded into five consecutive laminin G-like (LG) domains. A major function of laminins is to form cell-associated polymers, which provide structural support as well as platforms for signalling [1], [2]. Cell attachment is mediated by the LG domains and the very C-terminus of the coiled coil, whereas polymer formation is mediated by the tips of the three short arms [6].

The three short arms of the laminin heterotrimer are composed of long repeats of laminin-type epidermal growth factor-like (LE) domains, capped by laminin N-terminal (LN) domains that mediate the self-interaction of laminins. The LE repeats are interrupted once (β and γ chains) or twice (α chains) by globular domains of unknown function (Fig. 1). The inserted domains in the β and γ chains are called LF and L4, respectively [5] (or IVB and IVA in UniProt); the two types are not related at the sequence level. In the α1 and α2 chains, both inserts are L4 domains, whereas in the α3 and α5 chains only the second insert domain is an L4 domain. The first insert in α3 and α5 is much longer (~ 580 residues) and has weak homology to the LF domain in the first ~ 200 residues.

The LE fold was first described by Stetefeld et al. [7], who determined the crystal structure of LE domains 7–9 of the laminin γ1 chain (we follow the UniProt convention of numbering the LE domains consecutively from the N-terminus; Stetefeld et al. termed this region γ1III3–5). Subsequent structures of laminin short arm tips [8], [9], netrins [10], [11], [12], and netrin G proteins [13], [14] have confirmed that LE domains are largely devoid of secondary structure and are lacking a conventional hydrophobic core. Instead, the LE domain is held together by eight conserved cysteines, which are linked 1–3, 2–4, 5–6 and 7–8. This disulphide bonding pattern creates four loops: loop a (Cys1–Cys3), loop b (Cys2–Cys4), loop c (Cys5–Cys6), and loop d (Cys7–Cys8). The L4 domains in laminin α and γ chains are inserted between cysteines 3 and 4 of a standard 8-cysteine LE domain. The LF domains in laminin β chains, and the large inserts in α3 and α5 chains, are inserted after a truncated LE domain lacking the 7–8 disulphide bond (LE5 in β chains).

A recent crystal structure of the second L4 domain of the laminin α2 chain (α2 L4b) revealed an irregular β-sandwich similar to bacterial carbohydrate binding modules, ephrin-binding modules and MAM domains [15]. In the present study, we report that the LF domain of the laminin β2 chain has a similar fold, despite sequence identity of only ~ 10%, but that the LF domain additionally contains a unique α-helical region that makes extensive interactions with the preceding LE domain, LE5. Although separated by a 220-residue insert, LE5 and LE6 are observed in the rod-like arrangement that is typical of tandem LE domains. Thus, the globular LF domain in laminin β short arms is accommodated without interrupting the regular array of LE domains.

## Results

### Crystal structure of laminin β2 LE5-LF-LE6

We produced a panel of recombinant laminin fragments containing the β1 or β2 LF domain flanked by one or more LE domains for crystallisation trials. Crystals of the laminin β2 LE5-LF-LE6 fragment were suitable for structure determination. The β2 LE5-LF-LE6 structure was solved by the multiple isomorphous replacement method and refined to R_free_ = 0.210 at 1.85 Å resolution (Table 1). The asymmetric unit of the monoclinic crystals contains two structurally very similar copies of β2 LE5-LF-LE6 (r.m.s. deviation of 0.62 Å for 301 Cα atoms). The β2 LE5-LF-LE6 structure is complete except for residues 705–712 in the LF domain, which are presumed to be disordered.

**Fig. 2:**
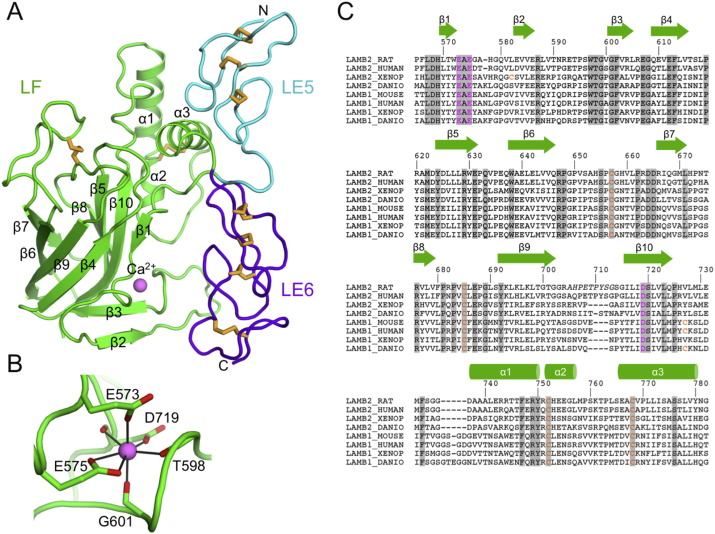
Crystal structure of laminin β2 LE5-LF-LE6. (A) Cartoon representation. Disulphide bonds are shown as orange sticks. A Ca^2 +^ ion is shown as a pink sphere. The secondary structure elements of the LF domain are labelled. (B) Details of the Ca^2 +^ coordination in the LF domain. (C) Sequence alignment of selected LF domains. Cysteines and residues involved in Ca^2 +^ binding are shown in orange and pink, respectively. The sequence numbering and secondary structure elements of the rat β2 LF domain are shown above the alignment.

The β2 LE5-LF-LE6 fragment has a surprisingly compact structure with approximate dimensions of 60 Å × 50 Å × 40 Å (Fig. 2A). The LE5 and LE6 domains are aligned with their long axes on one side of the structure. Even though LE5 and LE6 are separated by 220 residues in sequence, they interact similarly to consecutive LE domains in laminin short arms (see below for details). The 220 residues inserted between LE5 and LE6 are folded into two distinct regions, a 10-stranded β-sandwich (residues 564–726) and an α-helical region (727–783). To be consistent with the established nomenclature [5], we refer to the entire insert as the LF domain.

The β-sandwich of the LF domain consists of two antiparallel sheets of complicated topology, β1–(β2–β3)–β10–β5–β8 and β4–β9–β6–β7. The long central strand β10 interacts with β1 and with β3. A disulphide bond, Cys657–Cys685, links the β6–β7 and β8–β9 loops. Strong spherical electron density indicated that a metal ion is bound to the β1–β2 and β2–β3 loops. This ion was assigned as Ca^2 +^ based on the nature of its ligands, the coordination geometry, and ion-ligand distances of 2.4–2.5 Å. The Ca^2 +^ ion is coordinated by the side chains of Glu573 (monodentate), Glu575 (bidentate) and Asp719 (monodentate), as well as the carbonyl oxygen atoms of residues 598, 601 and 719; the resulting coordination geometry is a pentagonal bipyramid (Fig. 2B). Sequence comparison predicts that the Ca^2 +^ ion is present in all LF domains (Fig. 2C).

The α-helical region of the LF domain consists of three helices held together by a disulphide bond, Cys752–Cys768, and a substantial hydrophobic core (Val727, Leu730, Met732, Phe733, Phe747, Leu771, Leu772, Ala775, Val779) that is conserved in other LF domains (Fig. 2C). Helix α3 plays an important role in mediating the apparently stable association of the LF domain with LE5: α3 residues Ser774 and Ser776 interact with loop a of LE5 (residues 528–531), and Gly782 and Val783 interact with loop c of LE5 (residues 547–550). In contrast, the LF domain makes only one tenuous contact with LE6, and there is a large solvent-filled cavity between the two domains (Fig. 2A). Altogether, the interface between the LF domain and LE domains 5 and 6 buries ~ 1600 Å^2^ of solvent-accessible surface.

**Fig. 3:**
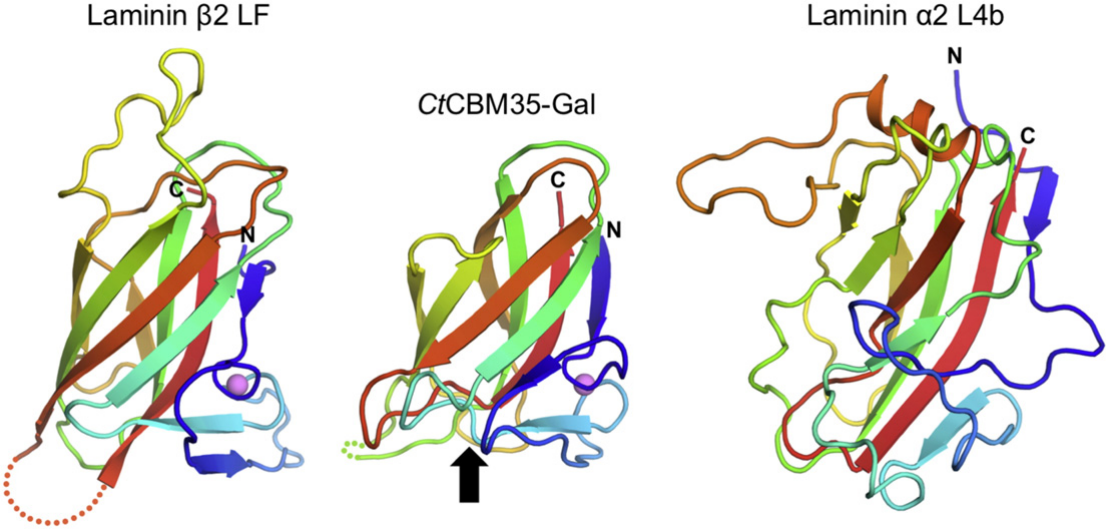
Comparison of the laminin β2 LF domain (β-sandwich only) with a CBM35 from *Clostridium thermocellum*[17] and the laminin α2 L4b domain [15]. The polypeptide chains are rainbow-coloured from blue (N-terminus) to red (C-terminus). The arrow indicates the location of the galactose binding site in *Ct*CBM35-Gal.

A search for related structures using PDBeFold [16] revealed that the β-sandwich of the LF domain is related to the family 35 carbohydrate binding module (CBM35) structure, despite pairwise sequence identities of just over 10% (Fig. 3); the α-helical region of the LF domain has no counterpart in CBM35 or any other protein. The galactose-binding CBM35 of a cell wall-degrading enzyme of *Clostridium thermocellum* [17] can be superimposed onto the LF domain with a r.m.s. deviation of 2.2 Å for 107 aligned Cα atoms (Z-score of 8.0 in PDBeFold), and even the Ca^2 +^ site is conserved in the two domains. The loops connecting the strands of the β-sandwich are quite different, however, and the carbohydrate binding site of CBM35 is not conserved in the LF domain. Comparison of the laminin β2 LF domain with the laminin α2 L4b domain [15] shows that the two domains share a β-sandwich of the same topology, but are otherwise not obviously related (Z-score of 3.4 in PDBeFold).

**Fig. 4:**
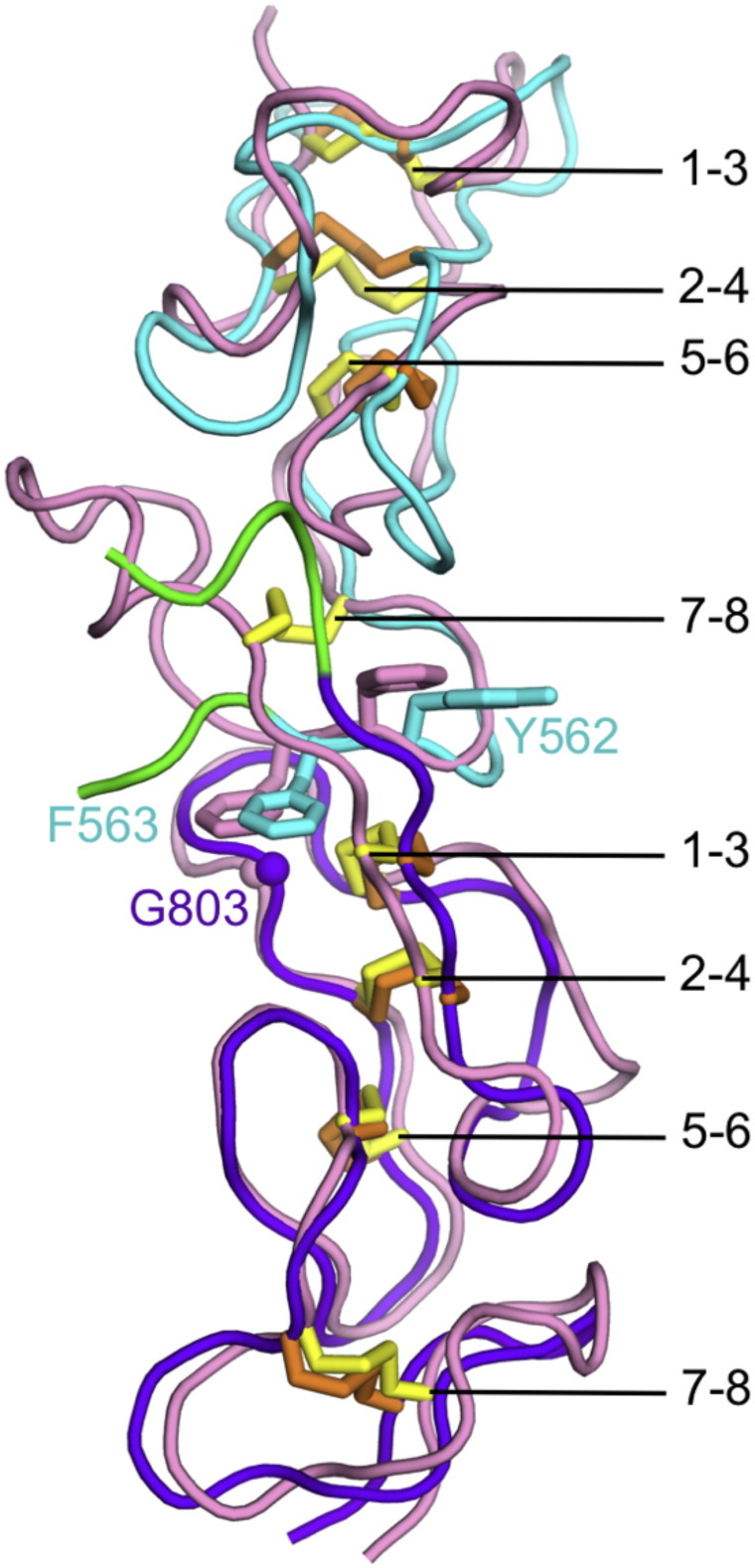
Superposition of laminin β2 LE5 and LE6 (this work) onto laminin γ1 LE8–LE9 [7] using the 14 cysteines that are common to both structures (r.m.s. deviation of 1.67 Å for 84 atoms). LE5 and LE6 of laminin β2 are shown in cyan and purple, respectively. The site of the LF insertion is in green. LE8 and LE9 of laminin γ1 are in pink. The disulphide bonds in the β2 and γ1 chains are shown as orange and yellow sticks, respectively, and are labelled. Selected residues involved in LE interdomain interactions (see text) are shown in sticks and are labelled.

An unexpected feature of the laminin β2 LE5-LF-LE6 structure is the relative arrangement of LE5 and LE6, which closely resembles the canonical arrangement of LE domains that are not separated by an inserted domain. A superposition of the β2 LE5-LF-LE6 structure with domains LE8–LE9 of the laminin γ1 chain [7] is shown in Fig. 4. The seven disulphide bonds common to the two structures are closely matched, but β2 LE5 lacks the 7–8 disulphide bond of γ1 LE8 to accommodate the inserted LF domain. Stetefeld et al. [7] observed that the consecutive LE domains in γ1 LE7–9 interact in a stereotypical manner, with loop d of the N-terminal LE domain packing against the 1–3 disulphide bond and loop b of the C-terminal LE domain. Of particular importance are a pair of aromatic residues in loop d and a glycine loop b. Remarkably, the interaction between these elements is conserved in β2 LE5-LF-LE6 (Tyr562 and Phe563 in LE5, Gly803 in LE6; Fig. 4). Thus, the LF domain is inserted into the short arm of laminin β chains without interrupting the regular array of LE domains.

### Solution structure of laminin β1 LN-LE6

**Fig. 5:**
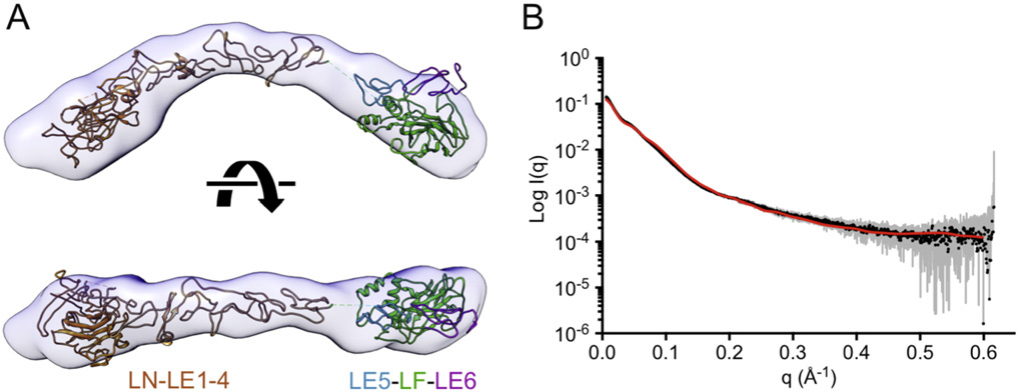
SAXS structure of laminin β1 LN-LE6. (A) *Ab initio* envelope and fitted cartoon models of the laminin β1 LN-LE4 structure [8] and the LE5-LF-LE6 structure (this work). Residues 494–512 are missing from the atomic models, leaving a gap of 30 Å (broken line). (B) Fit of the scattering curve calculated from the atomic model shown in A (red curve) to the experimental SAXS data (black dots with grey error bars).

Having determined crystal structures of the LN-LE4 region [8] and LE5-LF-LE6 region (this work) of laminin β chains, we were interested in how these two regions are connected in the β chain short arm. We therefore produced a laminin β1 LN-LE6 protein and determined its structure in solution using small-angle X-ray scattering (SAXS) (Fig. 5). The β1 LN-LE6 envelope obtained by *ab initio* reconstruction resembles a boomerang with bulbous tips. The β1 LN-LE4 crystal structure [8] can be fitted to either arm of the boomerang, with the LN domain occupying one bulbous tip; the remaining volume is matched reasonably well by the LE5-LF-LE6 crystal structure (Fig. 5A). Because the envelope has a nearly symmetrical shape, there is an ambiguity in placing the atomic structures. The β1 LN-LE4 placement shown in Fig. 5A has a slightly better fit to the envelope than the alternative one (correlation of 0.66 compared with 0.64; 10% fewer atoms outside the envelope). A scattering curve back-calculated from the atomic model fits the SAXS data well over the entire q range, with a χ^2^ value of 5.6 (Fig. 5B).

The low-resolution structure of β1 LN-LE6 determined by SAXS shows a more pronounced curvature in the LE tandem than would be predicted if all LE domains interacted in the canonical manner described above. The rigid-body fitting of the LN-LE4 and LE5-LF-LE6 crystal structures suggests a bend between LE4 and LE5, but the low resolution of the SAXS structure precludes the accurate modelling of this deviation from a straight arrangement. Interestingly, the recombinant protein used to determine the β1 LN-LE4 crystal structure [8] actually included the LE5 domain, but LE5 and the C-terminus of LE4 were disordered in the crystals, suggesting that the LE4–LE5 junction might be flexible.

## Discussion

The different domain types present in laminin chains have distinct functions: the α-helical regions are required for coiled coil formation leading to heterotrimer formation; the LN domains are required for the self-interactions leading to network formation; the LG domains are required for interactions with cellular receptors; the γ1 LE8 domain is required for nidogen binding; and the LE repeats more generally are believed to function as network spacers [6]. Curiously, no function has yet been assigned to the L4 or LF domains that occasionally interrupt the LE repeats, even though their conservation in all laminin genes suggests an important function [3]. The LF and L4 domains are not obviously related at the sequence level, but our crystal structure of the β2 LF domain (Fig. 2) and a recently reported structure of the α2 L4b domain [15] now show that the two domain types share a common CBM35-like fold (Fig. 3). Structure prediction using Phyre2 [18] indicates that the N-terminal third of the unique insert within the α3/α5 chains also adopts this fold (not shown). There is no indication, however, that LF or L4 domains bind carbohydrates, and the function of these inserted domains remains enigmatic.

Genetic excision would be one way to decipher the function of LF and L4 domains, but their internal location within laminin chains makes it difficult to design a clean excision strategy. We show here that the LF domain is inserted into the laminin β chain without disrupting the regular interdomain packing of LE domains (Fig. 4), and a similar situation is predicted for the more common L4 domains [15]. Thus, it may be feasible to construct laminin short arms of unaltered length that consist solely of LE domains. Such artificial constructs might be less susceptible to proteolysis than the mutated α2 chain in patients with muscular dystrophy, which have a 63-residue deletion in the α2 L4b domain [19]. Experiments with non-natural laminin variants have shown that network formation does not appear to be very sensitive to the exact architecture of the short arms, so long as the laminin heterotrimer contains a full complement of α, β and γ LN domains [20], [21]. It seems reasonable to assume that network formation requires a certain flexibility of the short arms, in order to allow the three LN domains to interact optimally [22]. A low-resolution structures of the entire γ1 short arm revealed a bend at the presumed position of the L4 domain [23], and the β1 LN-LE6 region is also markedly curved in solution (Fig. 5). Whether these deviations from a straight LE tandem have a functional significance, or indeed impart flexibility, is unclear.

The LF domains of the laminin β1 and β2 chains were studied biochemically by Sasaki et al. [24]. In the β1 LF domain produced in HEK293 cells, a single chondroitin sulphate chain was attached to Ser721. This residue maps to the loop connecting strand β10 to helix α1 in our structure (Fig. 2). The β1 LF domain also contains an unpaired cysteine, Cys710, which caused dimer formation of the recombinant LF protein [24]. The corresponding residue in the β2 LF domain, Val727, is buried in the hydrophobic core between helices α1–α3, which interact with the LE5 domain (Fig. 2). The lack of LE5 in the β1 LF construct studied by Sasaki et al. may have destablised the α-helical region, thereby exposing the unpaired cysteine for disulphide bond formation.

In summary, our structure of the LE5-LF-LE6 region of the laminin β2 chain has uncovered a structural relationship between the two types of globular domains that are found in laminin short arms, the L4 and LF domains. Moreover, the LF domain is found to be inserted in such a way that the LE5 and LE6 repeats interact as if they were contiguous in sequence. These findings have implications for the evolution of laminins and may help in identifying the elusive function of the LF domains in laminin β chains.

## Materials and methods

### Expression vectors

The coding sequence for the LE5-LF-LE6 region of rat laminin β2 (UniProt P15800) was amplified from a cDNA kindly provided by Jeffrey H. Miner. The PCR product was cloned into a modified pCEP-Pu vector [25]. The pCEP-encoded protein consists of the BM-40 signal peptide, followed by a hexahistidine tag, a tobacco etch virus (TEV) protease cleavage site, and laminin β2 residues 523–833. The N-terminus of the protein after TEV protease cleavage includes a vector-derived GALA sequence. DNA sequencing of the rat laminin β2 construct revealed that residue 545 is arginine (as in mouse laminin β2), and not proline as in UniProt P15800.

The coding sequence for the LN-LE6 region of mouse laminin β1 (UniProt P02469) was amplified from a cDNA kindly provided by Peter D. Yurchenco. The PCR product was cloned into a modified pCEP-Pu vector [25]. The pCEP-encoded protein consists of the BM-40 signal peptide, followed by laminin β1 residues 22–820, and a C-terminal hexahistidine tag (AAAHHHHHH). The N-terminus of the mature protein includes a vector-derived APLA sequence.

### Protein expression and purification

The laminin β2 LE5-LF-LE6 protein was produced using the FreeStyle™ 293 Expression System (ThermoFisher Scientific) following the manufacturer's procedures. Briefly, 293-F cells were grown in a shaking incubator at 37 °C with 8% CO_2_ in serum-free FreeStyle™ 293 Expression medium to a cell density of 10^6^ cells/ml. The cells were transfected with the expression vector using polyethylenimine (PEI; VWR International) using a *w*/*w* ratio of DNA:PEI of 1:3. The conditioned medium containing the secreted protein was collected 72 h after transfection. The filtered cell culture supernatant was adjusted to a final concentration of 20 mM Na-HEPES (pH 7.5) and loaded onto a 5-ml HisTrap Excel column (GE Healthcare) using an Äkta Purifier (GE Healthcare). The column was washed with 20 mM Na-HEPES, 150 mM NaCl (pH 7.5) and the protein was eluted with the same buffer containing 200 mM imidazole. Fractions containing protein were concentrated using a Vivaspin centrifugal device (Sartorius) to a final concentration of 1 mg/ml. The protein solution was dialysed and digested with His-tagged TEV protease (made in *Escherichia coli* using an expression vector kindly provided by Stephen Curry) in a enzyme:substrate ratio of 1:10 for 18 h at 4 °C. The reaction mixture was loaded onto a 1-ml HisTrap FF column (GE Healthcare) and the flow through was collected. Fractions containing protein were concentrated using a Vivaspin centrifugal device and further purified on a Superdex 200 10/300 column (GE Healthcare) using 20 mM Tris–HCl, 150 mM NaCl (pH 7.5) as the running buffer.

The laminin β1 LN-LE6 protein was produced in human embryonic kidney 293 c18 cells and purified as described for other laminin short arm fragments [9].

### Crystallisation

Screening was done at 20 °C by the sitting-drop vapour diffusion method using 96-well plates (Greiner) and a range of commercial screens. A Mosquito nanolitre robot (TTP Labtech) was used to set up 200 nl drops. The initial crystals of laminin β2 LE5-LF-LE6 were obtained using a protein concentration of 10 mg/ml and condition H4 of the Morpheus screen (Molecular Dimensions), which contains a mixture of amino acids and polyethylene glycols buffered at pH 6.5. Larger crystals were grown in 2 μl hanging drops using the same precipitant solution. The crystals first appeared as heavily intergrown plates, which over the course of a week matured into large single crystals. Heavy-atom derivatives were prepared by soaking the crystals for 18 h in 0.2 mM UO_2_(NO_3_)_2_ or 0.5 mM p-chloromercuribenzoic acid (PCMB), or for 30 s in 500 mM NaI. The crystals were flash-frozen in liquid nitrogen directly from the drops.

### Crystal structure determination

Diffraction data were collected at 100 K at beamlines I04-1 (native data) and I03 (derivative data) of the Diamond Light Source (Oxfordshire, UK). The data were processed using XDS [26] and programs of the CCP4 [27] suite as implemented in the XIA2 pipeline [28]. The crystals were found to belong to space group *C*2 with two copies of laminin β2 LE5-LF-LE6 in the asymmetric unit. CC_1/2_ was used to determine the resolution limits [29]. The phases were determined by multiple isomorphous replacement with anomalous scattering (MIRAS) using AutoSol as implemented in the PHENIX suite [30]. Manual rebuilding and refinement were done using COOT [31] and PHENIX. The figures were generated using PyMOL (www.pymol.org).

### Small-angle X-ray scattering analysis

The laminin β1 LN-LE6 protein was concentrated to 9 mg/ml in 20 mM Na-HEPES, 150 mM NaCl, 2 mM CaCl_2_ (pH 7.5) and analysed by small angle X-ray scattering with in-line size exclusion chromatography (SEC-SAXS) at the SWING beamline of the SOLEIL synchrotron (Paris, France). The chromatographic separation of the sample was achieved using an Agilent HPLC with a BioSEC-3 column (300 Å pore size). SAXS data were recorded over a momentum transfer range of 0.0063–0.6162 Å^− 1^. The data were integrated, buffer-subtracted, and merged using the FOXTROT software [32]. PRIMUS [33] was used to obtain R_G_ values *via* Guinier analysis. GNOM [34] was used to obtain D_max_ values and generate a P(r) distribution for *ab initio* envelope determination, which was done using DAMMIF/DAMINN [35]. A total of 20 DAMMIF envelopes were averaged and refined to convergence using DAMMIN. The crystal structure of laminin β1 LN-LE4 [8] and a homology model of laminin β1 LE5-LF-LE6 (created from the laminin β2 LE5-LF-LE6 crystal structure using Phyre2 [18]) were fitted to the refined envelope using simultaneous feature-based docking in SCULPTOR [36]. Comparison of the atomic model with the raw scattering data was done using FOXS [37]. The figures were generated using UCSF Chimera (www.cgl.ucsf.edu/chimera).

## Database reference

The coordinates of the laminin β2 LE5-LF-LE6 structure have been deposited in the Protein Data Bank under code 5LF2.

## Figures and Tables

**Table 1:** Crystallographic statistics.

	Native	UO_2_(NO_3_)_2_	PCMB	KI
*Data collection*				
Beamline	Diamond I04-1	Diamond I03	Diamond I03	Diamond I03
Wavelength (Å)	0.920	0.976	0.976	0.976
Resolution range (Å)	62.9–1.85 (1.90–1.85)	70.2–1.84 (1.89–1.84)	95.7–1.98 (2.03–1.98)	69.9–2.29 (2.35–2.29)
Space group	*C*2	*C*2	*C*2	*C*2
Unit cell dimensions				
*a*, *b*, *c* (Å)	144.32, 55.35, 111.34	142.88, 54.69, 109.40	142.19, 54.75, 109.23	142.33, 54.79, 109.06
α, β, γ (°)	90, 119.38, 90	90, 119.17, 90	90, 118.82, 90	90, 118.92, 90
Unique reflections	65,011	48,727	48,638	33,179
Multiplicity	6.8 (7.1)	4.5 (2.5)	3.0 (2.1)	4.8 (4.3)
Completeness (%)	99.1 (99.2)	76.0 (25.1)	94.2 (67.3)	99.2 (99.1)
Mean I/σ(I)	15.7 (1.4)	14.8 (1.5)	10.5 (1.3)	8.4 (1.6)
CC_1/2_	0.999 (0.635)	0.998 (0.468)	0.996 (0.531)	0.991 (0.489)
R_merge_	0.057 (1.29)	0.059 (0.680)	0.071 (0.667)	0.149 (0.864)
R_deriv_		0.170	0.217	0.348

*Refinement*				
Protein atoms	4567			
Solvent atoms	435			
R_work_	0.178			
R_free_	0.210			
R.m.s.d. bonds (Å)	0.014			
R.m.s.d. angles (°)	1.28			
Ramachandran plot				
Favoured (%)	98.0			
Allowed (%)	2.0			
Outliers (%)	0			
